# Letter from Dr. R. G. Williams; Sweating Feat

**Published:** 1890-02

**Authors:** R. G. Williams

**Affiliations:** Whitney, Texas


### SWEATING FEET.

WHITNEY, TEXAS, February 3, 1890.

*Editor Daniel's Texas Medical Journal:*

I see extensively copied from journal to journal, the article on "Sweating of feet," wherein is recommended chromic acid. Let me state I have found nothing comparable to tannic acid. This should be freely applied after each washing of feet. Tannic acid produces discoloration of the parts, but that is of little moment. It is most effective in preventing sweating, and overcomes all odor.

R. G. WILLIAMS, M. D.

